# Promoting psychological resilience during the perinatal period with a digital tool (the HealthyMoms4MentalHealth-app): a study protocol for a randomised controlled trial in Sweden

**DOI:** 10.1186/s12889-026-26804-8

**Published:** 2026-10-03

**Authors:** Marie Löf, Katarina Ekelöf, Christine Delisle Nyström, Gunilla Lönnberg, Brjánn Ljótsson, Gerd Almqvist Tangen, Caroline Lilliecreutz, Kristin Thomas

**Affiliations:** 1 Department of Medicine, Karolinska Institutet, Huddinge, Stockholm, Sweden; 2 Center Clinical Research, Skånes University Hospital, Lund, Sweden; 3 Department of Public Health and Caring Sciences, Social Medicine/CHAP, Uppsala University, Uppsala, Sweden; 4 Department of Pediatrics, Halland Hospital Halmstad, Halmstad, Sweden; 5 Department of Pediatrics, Institute of Clinical Sciences, Sahlgrenska Academy, University of Gothenburg, Gothenburg, Sweden; 6 Region Östergötland, Department of Obstetrics and Gynecology, Linköping, Sweden; 7 Department of Biomedical and Clinical Sciences, Linköping University, Linköping, Sweden; 8 Department of Health Medicine and Caring, Linköping University, Linköping, Sweden

**Keywords:** Randomized controlled trial, Perinatal mental health, Digital intervention

## Abstract

**Background:**

Pregnancy and childbirth are major life transitions that can significantly impact women’s mental health, with depression and anxiety being the most commonly reported. International and Swedish studies estimate that up to one in four women experience anxiety or depressive symptoms during pregnancy or postpartum. Poor maternal mental health is associated with adverse outcomes for both mother and child, including preterm birth and low birth weight. Psychological resilience has emerged as a key protective factor, linked to reduced psychological distress and improved emotional regulation during pregnancy. Despite the potential of scalable digital interventions, few have been scientifically evaluated or tailored to the perinatal period.

**Objectives:**

To estimate the effectiveness of a new digital intervention (the HealthyMoms4MentalHealth-app) on psychological resilience among women during the perinatal period.

**Methods:**

The study is a two-arm parallel-group randomized controlled trial. A total of 440 pregnant women will be randomized to either the intervention group (HealthyMoms4MentalHealth-app + standard care) or control group (active control-app + standard care). Outcomes will be assessed at baseline prior to randomization (12–14 gestational weeks), and follow-ups in late pregnancy (35–37 gestational weeks), and postpartum (8–9 weeks). The primary outcome will be psychological resilience and secondary outcomes include psychological wellbeing and depressive symptoms. Exploratory outcomes will include fear of childbirth, self-compassion, pre- and postnatal attachment and health behaviours. The intervention was developed using a human-centred design approach and is grounded in positive psychology, self-compassion, and empirical data from qualitative interviews with perinatal care professionals. The intervention begins between gestational weeks 12–14 and continues until 8 weeks postpartum and is aimed to be a universal, health promotive and upstream digital intervention targeting a large proportion of the perinatal population. The active control app include content on healthy eating, healthy weight gain and physical activity during pregnancy.

**Conclusion:**

This trial will provide evidence on the effectiveness of a digital mental health intervention tailored to the perinatal period. Findings may inform future strategies for scalable, accessible health promotive mental health support for pregnant and postpartum women.

**Trial registration:**

The trial has been registered at ClinicalTrial.gov: trial identifier NCT06862193; trial date 5th March 2025. Full protocol and statistical analysis plan will be available with the trial’s registration record.

**Protocol version:**

3.0, 3rd November 2025.

**Supplementary Information:**

The online version contains supplementary material available at https://doi.org/10.1186/s12889-026-26804-8.

## Introduction

Pregnancy and childbirth are significant life events that can impose difficult challenges on women’s physical and mental health. Pregnancy, and the transition to parenthood, is marked by hormonal changes, physical transformations, the shifting of social roles, and increased psychological demands, all of which can contribute to major emotional strain for the pregnant woman [1]. These adjustments may contribute to increased vulnerability of developing mental health problems during the perinatal period (pregnancy and postpartum) with depression and anxiety being the most common reported conditions [2–5]. A meta-analysis of 102 studies across 34 countries estimated the prevalence of anxiety during pregnancy to range between 15.2% and 24.6%, with anxiety postpartum affecting approximately 15% of women [6]. International studies suggest that 7–16% of women experience depressive symptoms during pregnancy [7–9] and 9–12% postpartum [10]. Swedish cohort studies report comparable findings, with depression affecting around 13% of women [11, 12] and anxiety around 15% of women [13] during pregnancy. Longitudinal data has identified distinct depressive trajectories, with 14.6% of women experiencing chronic symptoms and smaller subgroups developing early (10.9%) or late (5.4%) postpartum-onset depression [14]. Comorbid anxiety and depression are also common, affecting approximately 9% of women during the perinatal period [15]. Key risk factors for developing mental health problems during the perinatal period include a history of mental illness, low social support, stressful life events and socioeconomic disadvantage such as unemployment or low income [5, 16–18].

Poor maternal mental health and adversity are associated with adverse outcomes for both mother and child. Elevated stress and anxiety have been linked to infants being less emotionally stable, such as having lower levels of emotional self-regulation [19]. Furthermore, a population-based study [20] and meta-analysis (*n* = 88) [21] showed that prenatal stress and poor maternal mental health increase the risk for adverse birth outcomes such as preterm birth and low birth weight in infants. Interestingly, a longitudinal study assessing the impact of positive antenatal mental health during pregnancy showed that this was associated with improved cognitive, social, and emotional development of the child up to 24 months after birth [22]. These findings underscore the importance of promoting maternal mental health throughout the perinatal period. One potential pathway to achieving this lies in strengthening protective psychological factors that can buffer against stress and adversity. In this context, resilience—a key determinant of adaptive functioning under challenging circumstances—has emerged as an important focus of research [23–26].

Resilience, broadly defined as the capacity to adapt positively in the face of adversity, may play a critical role in supporting psychological well-being during pregnancy and post-partum for the woman. Indeed, resilience and resilience-enhancing methods (e.g., emotional regulation) have been linked with reduced symptoms of anxiety and depression in general populations [24, 27]. As pregnancy involves profound physiological, emotional, and social changes [2], resilience has been proposed as a key protective factor, with research demonstrating associations between higher resilience levels and lower levels of depression, anxiety, and perceived stress during pregnancy [23, 28]. Resilient individuals are more likely to employ adaptive coping strategies and maintain emotional stability [29–31], which can buffer the impact of common stressors during pregnancy, such as fears related to childbirth, physical discomfort, or changes in social roles.

Maternity health care in Sweden is the main healthcare provider during pregnancy whereby health promotion is one key area. However, evidence-based tools are lacking, and reports show that midwives struggle to incorporate preventative work in routine care [32]. Digital interventions (e.g., mobile apps) have shown to be promising cost-effective tools in mental health promotion [33]. Although there is a large number of pregnancy health apps available on the market, few have been scientifically evaluated [34]. Furthermore, within digital health research, the majority of interventions have been developed and evaluated in adult general populations rather than tailored for the perinatal period [35]. There is research on digital mental health interventions among pregnant women; however, most interventions target mental health problems, such as depressive symptoms, rather than aiming to promote mental health through e.g., resilience [36, 37].

Few studies have explored perinatal mental health using a salutogenic framework (i.e., by focusing on strengths, resilience and resources for health). One quasi-experimental study (*n* = 74) reported improvements in self-reported well-being and reductions in psychological distress among users of a mobile self-help intervention (mindfulness, savouring the moment) [38]. Another pilot study demonstrated high user acceptability and qualitative evidence of enhanced emotional awareness and perceived support among pregnant women (*n* = 88) [39]. A non-randomized pilot evaluating a supported online resilience-enhancing intervention for pregnant women found increased resilience, improved emotional well-being, and positive user engagement [40]. Similarly, a pilot randomized controlled trial (*n* = 367) [41] showed that a self-guided digital intervention strengthened positive psychological functioning (e.g., self-compassion, life satisfaction) and reduced depressive symptoms, suggesting benefits even for non-clinical populations. Together, these studies indicate improvements in well-being, reductions in distress and favorable engagement. Although these preliminary findings show promising results and a potential for digital salutogenic interventions, rigorous trials with larger samples and extended follow-up periods are needed to establish their effectiveness.

This protocol describes a randomised controlled trial with the overall aim to investigate the effectiveness of a new digital intervention, the HealthyMoms4MentalHealth-app (HM4MH) on mental health outcomes among women during the perinatal period. Specifically the effectiveness on:

- psychological resilience (primary outcome) as well as depressive symptoms and psychological wellbeing (secondary outcomes).
- exploratory outcomes during pregnancy and postpartum (self-compassion, parental bonding during pregnancy and postpartum, fear of childbirth and health behaviours).
- obstetric and neonatal outcomes.

## Methods and analysis

### Design

**Fig. 1:**
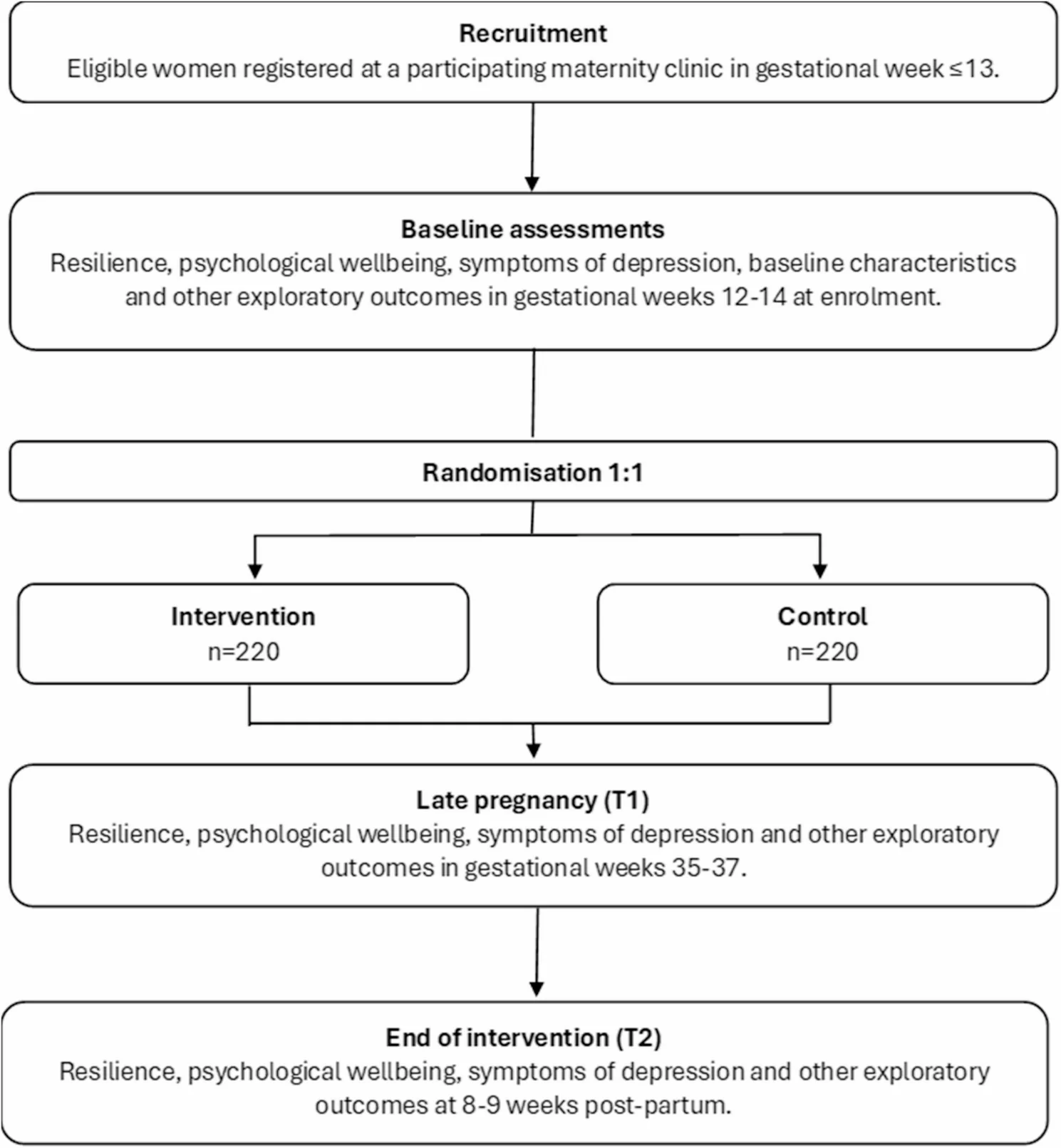
Study overview for the HM4MH-trial

A two-arm parallel group randomised controlled trial will be conducted to investigate the effectiveness of a digital intervention (the HM4MH-app) on mental health outcomes among women during the perinatal period. Women will be allocated to intervention (HM4MH-app targeting mental health) or active control group (focusing on lifestyle behaviours). The baseline assessments will be conducted prior to randomization. The women are randomized and the intervention is initiated in gestational week 12–14. Outcomes will be measured in late pregnancy (gestational week 35–37) and after birth (8–9 weeks post-partum) (Fig. 1). Objective (number and frequency of app-use) and subjective engagement data (in-app participant-reported ratings of app usability, satisfaction, and perceived helpfulness) will be monitored throughout. This protocol contains relevant items from the Standard Protocol Items: Recommendations for Intervention Trials 2013 statement [42] (Additional file 1). The Estimands framework (ICH E9 (R1) addendum) [43] was used when finalising study design (Additional file 2).

### Setting of the study

The project will be conducted within maternity health care in regions in the south of Sweden (Skåne, Stockholm, Jönköping and Kalmar). The regions have approximately 50 thousand births per year and encompass a mix of low- and high socio-economic areas as well as both urban and rural areas. Maternity health care in Sweden is integrated within a universal healthcare system, ensuring equitable access for all pregnant women (> 95% attendance). Services are provided free of charge and include comprehensive midwife-led antenatal care such as regular check-ups, medical screenings, and specialist consultations when indictated. Prenatal care begins early in pregnancy and comprises of eight to nine visits to a midwife with the first visit typically occuring between gestational weeks 6–12. Additional visits are scheduled throughout the pregnancy, with a focus on monitoring featal development, maternal health, and identifying potential risks or complications. Routine screenings include two ultrasounds, blood tests, and screening for conditions such as gestational diabetes, anemia, and preeclampsia. Pregnant women also receive information about nutrition, physical activity, mental health, and childbirth options. Postnatal care focuses on both maternal and infant health and is done in parallell by both maternal health care and child health care services. The first health visit for the newborn usually takes place within the first week of life, followed by routine checkups at child health care services. Postnatal care also includes mental health screenings for postpartum depression as well as parenting classes to learn about infant care, development, and safety.

### Comparator groups

#### Intervention

The intervention group will receive a digital intervention, the HM4MH-app targeting mental health, tailored to the perinatal period as well as standard maternity care (including regular monitoring of both maternal and fetal health parameters, such as blood pressure, blood glucose, ferritin levels, weight gain, symphysis–fundal height, fetal movements, and heart rate). The intervention was developed using a human-centred design approach involving end-users (pregnant women and mothers), researchers, practitioners and digital health experts (in preparation). The theoretical framework for the content in the HM4MH-app is based on positive psychology, self-compassion and empirical data from our qualitative interviews with healthcare professionals in the perinatal care system [44].

**Fig. 2:**
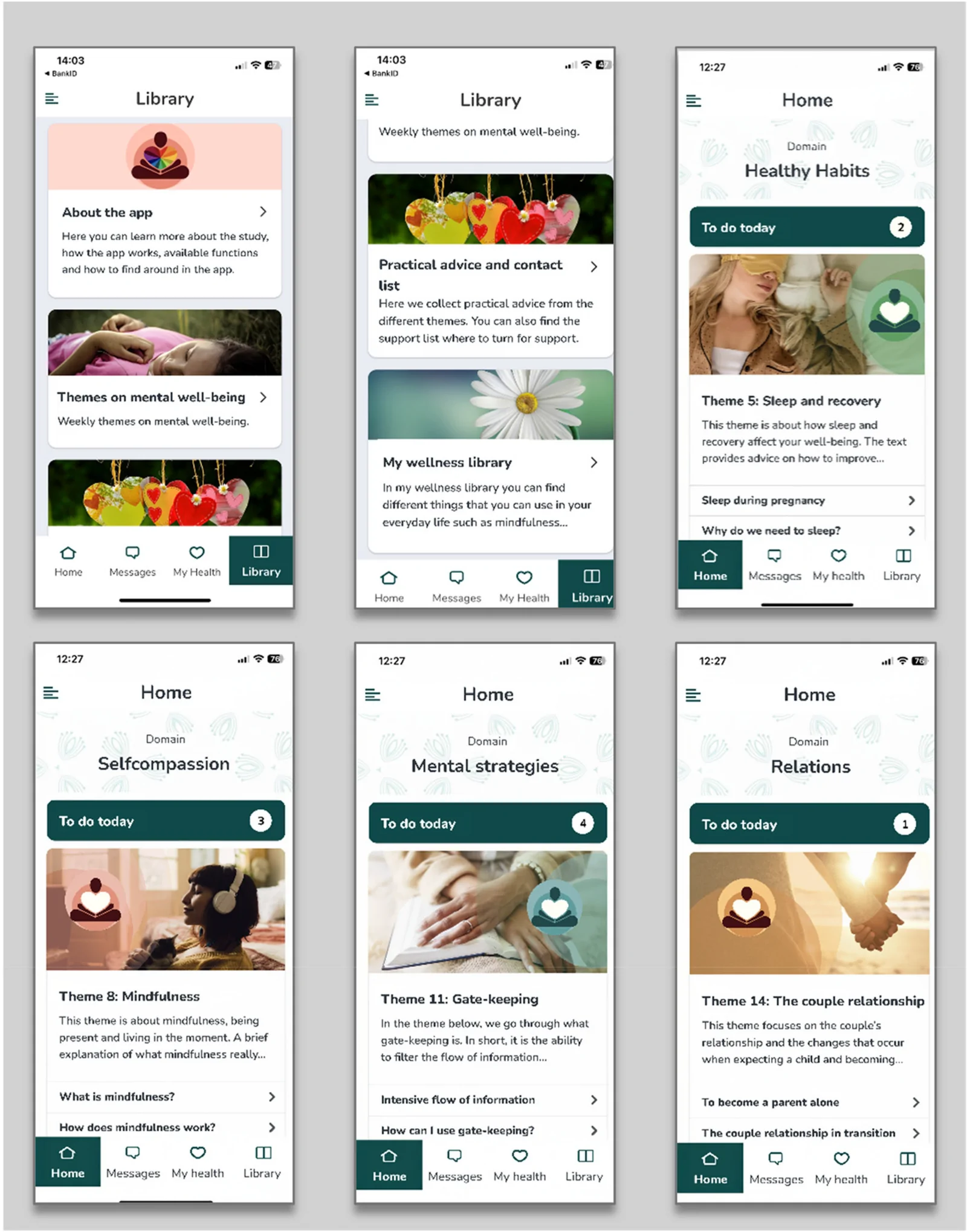
Examples of content of the intervention (the HM4MH-app)

The HM4MH-app is a multi-component intervention promoting mental wellbeing including content that aims to promote strategies for resilience. The content contains 33 educational themes from four different domains; self-compassion, mental strategies, relations and healthy habits. The themes are presented on a weekly basis together with 16 mindfulness exercises with focus on self-compassion, 20 reflection exercises and 12 training videos. The content is delivered via text, audio and visual modalities and runs from gestational week 12 to 8 weeks after birth. The HM4MH-app will be delivered via an online platform with a class II medicinal product approval (Cuviva AB). Figure 2 illustrates examples of content of the intervention.

#### Control group

The control group will gain access to a health app (HealthyMoms) tailored to pregnant women as well as standard maternity care (including regular monitoring of both maternal and fetal health parameters, such as blood pressure, blood glucose, ferritin levels, weight gain, symphysis–fundal height, fetal movements, and heart rate). The app is a theory-based behaviour change tool focused on promoting healthy eating, healthy weight gain and physical activity during pregnancy. The tool includes information, exercises and tips for healthy lifestyle habits and has been evaluated previously in a randomised controlled trial [45] showing beneficial effects on healthy weight gain and healthy lifestyle change. The control group will not have access to any content that aim to promote mental wellbeing. The HealthyMoms-app’s layout is similar and will be delivered via the same online platform (Cuviva AB).

#### Eligibility criteria

Eligibility criteria will be (i) pregnant women attending a participating maternity health care clinic who (ii) have access to their own mobile phone and BankID or FREJA ID (digital secure identification systems) and are (iii) randomised before the end of gestational week 14, (iv) adequate Swedish literacy to be able to provide informed consent and understand and use the app, that are (v) 18 years or older and have no history of psychiatric illness with psychosis.

#### Recruitment

Eligible pregnant women will be recruited at maternity healthcare clinics in the south of Sweden. Women will receive study information about the trial in several ways: (i) study information will be attached to the invitation that is sent by the maternity health care clinic before the first/registration visit (through paper-based or digital platforms), (ii) verbal and written study information will be given by midwifes during visit to the maternity health care centre, (iii) advertisement in maternity health care premises e.g., pamphlets or digital screens and (iv) through targeted social media informational campaigns. Women will register their interest in taking part in the study by completing an electronic registration form (name, telephone number, e-mail address and gestational week) which they access through QR-code or hyperlink enclosed in all advertisement material. All women will then be contacted by telephone by a researcher for more information about the study, eligibility screening and providing the opportunity to ask questions as well as initial informed consent. Informed consent will be obtained within the app via BankID or FREJA ID which are secure digital identification systems that allows the women to authenticate their identity and consent for participation online. Basline assessments are collected within the screening version of the app prior to adding the intervention (HM4MH) or active control (HealthyMoms) content of the app.

#### Randomisation

After baseline assessments, participants will be randomly assigned in a 1:1 ration to either intervention (HM4MH-app) or active control (HealthyMoms-app) group using a computer-generated allocation sequence. Randomisation will be stratified by parity (primiparous vs. multiparous) and by clinic site to ensure balanced group distribution.

#### Power calculations

Meta-analyses assessing the efficacy of resilience interventions in general populations [26, 46] including digital interventions [47] suggest small to moderate effect sizes on resilience (range corresponding to an approximate Cohen’s d of 0.3–0.5). Given the universal and salutogenic nature of the intervention, rather than a clinical one, changes that were considered psychologically meaningful for the women were used as the basis for calculations. A point-difference of approximately 2–3 points on the resilience measure (CD-RISC-10; range 0–40) may be deemed meaningful.

Thus, we aimed to detect a 2 points difference in the primary outcome assuming a standard deviation of 6 (unpublished data among pregnant women). This corresponds to a small effect size (approximately Cohen’s d = 0.30). Targeting 80% power, the required sample size was calculated to be 176 participants per group. Accounting for an anticipated attrition rate of 25%, the sample size was increased to 220 participants per group, resulting in a total sample size of 440 participants.

#### Outcomes

**Fig. 3:**
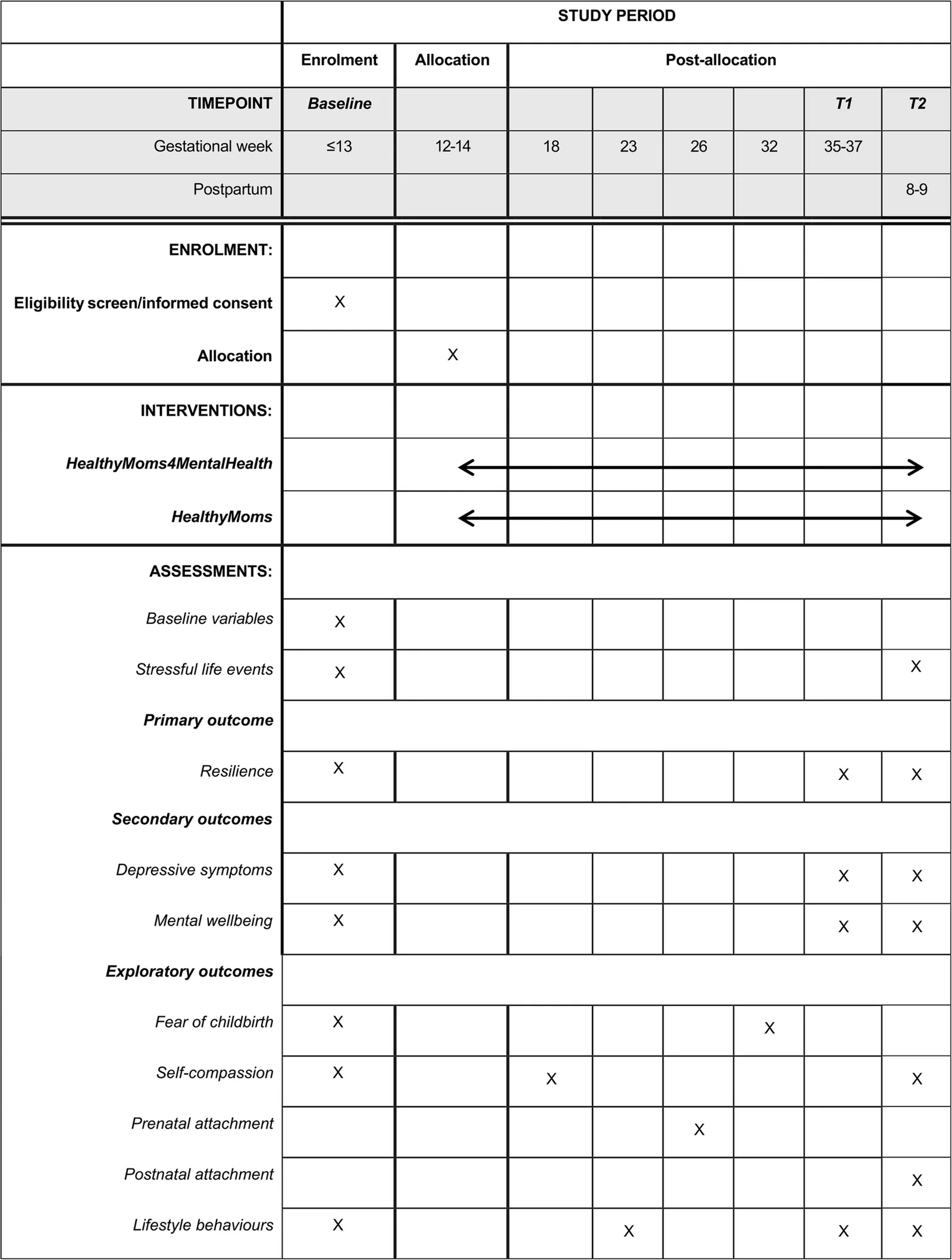
Participant timeline including enrolment, allocation and post-allocation

Primary outcome (resilience) and secondary outcomes (psychological wellbeing and depressive symptoms) will be measured at baseline (gestational weeks 12–14), timepoint 1 (gestational week 35–37) and timepoint 2 (8–9 weeks postpartum). At baseline we will also include questions regarding participant characteristics (e.g., age, employment, education, birth country, living situation and number of children and stressful life events). In addition, other exploratory outcomes will be measured at different time-points during pregnancy and post-partum (fear of childbirth, self-compassion, prenatal and parental attachment and lifestyle behaviours (Fig. 3). Data will be collected via questionnaires within respective app (HM4MH-app or HealthyMoms-app).

#### Primary outcome

Resilience will be measured using the Connor-Davidson Resilience Scale (CD-RISC-10) [19] which includes 10 items graded on a five-point scale from “not at all true” to “almost always true”. The items assess various aspects of resilience, such as emotional regulation, optimism, personal strength, and the ability to manage stress e.g., “I am able to adapt when changes occur” or “I tend to bounce back after illness or hardship”. Lower scores suggest lower resilience and possibly a greater vulnerability to stress and adversity whereas higher scores suggest higher resilience, meaning the individual is likely to be better equipped to handle stress or challenges. The CD-RISC-10 has shown high internal consistency (α = 0.85–0.91) and strong convergent validity with measures of depression, anxiety, and psychological well-being [48]. The CD-RISC-10 has been used in pregnant populations [49] and the longer 22 item version has been validated in non-clinical Swedish populations [50]. In analysis, total score (sum score of all items; range 0–40) will be used.

#### Secondary outcomes

Mental wellbeing will be measured with the Ryff Scales of Psychological Well-Being (RPWB) [51, 52], which includes 18 items. For this scale, higher scores indicate greater psychological well-being (range 18–108) and responses are given on a six-point Likert scale from “strongly disagree” to “strongly agree”. The scale measures multiple dimensions of well-being and includes six subscales: autonomy (independence, self-determination and resistance to social pressures), environmental mastery (ability to manage one’s life and surroundings), personal growth (realising one’s potential), positive relations with others (quality of interpersonal relationships), purpose in life (having direction and goals) and self-acceptance (positive self-regard). Both a composite score (sum score for all 18 items after accounting for reversed items) and scores for each subscale will be used in analysis. The scale has been validated in a Swedish population [52] and previously used in research among pregnant populations [53, 54].

Symptoms of depression will be measured using the Swedish version of the validated Edinburgh Postpartum Depression Scale (EPDS) [55, 56] which includes ten items graded on a four-point ordinal scale (0–3) with a score of 0-30 in which higher scores indicate more depressive symptoms. Participants rate the intensity of symptoms during the previous 7 days. The items on the scale focus on emotional and psychological symptoms, such as mood changes, feelings of guilt and anxiety. Clinically relevant cut-offs based on research in Swedish populations will be used in analysis: scores of ≥ 13 during pregnancy and ≥ 12 postpartum [12, 55].

#### Exploratory outcomes

Fear of childbirth will be measured using the Fear of Birth Scale (FOBS) [57] which includes a single item “How do you feel right now about the approaching birth?”. Women are asked to respond on two seperate scales ranging from (i) 0 = calm to 100 = worried and (ii) 0 = no fear to 100 = strong fear. The two scores are averaged to create a total FOBS-score. A mean score of ≥ 60 may be interpreted as significant fear of birth.

Self-Compassion Scale—Short Form (SCS-SF) [58] is a 12-item scale graded on a five-point scale from “almost never” to “almost always”. The scale measures a person’s level of self-compassion, which refers to the ability to treat oneself with kindness, understanding, and care, especially during times of suffering. Specifically, the scale measures six components of self-compassion: self-kindness, self-judgment, common humanity, isolation, mindfulness and over-identification. The SCS-SF has been validated in multiple languages and has been used among a pregnant population [59]. Total score (sum score of all items; range 12–60) will be used in analysis.

The Prenatal Attachment Inventory (PAI) [60] is a 21 item scale that assess the level of attachment as perceived by a woman during pregnancy. The inventory assess emotional, cognitive and behavioral aspects of the mother’s relationship with her unborn baby. Responses are given on a four-point likert scale ranging from almost never to almost always. Higher scores suggest stronger prenatal attachment and a positive emotional bond. The inventory has been validated in a pregnant population in Sweden [61]. Total score (sum score of all items; range 21–84) will be used in analysis.

Postpartum Bonding Questionnaire (PBQ) [62] is a 25 item scale with statements that are graded on a six-point likert scale from “Always” to “Never”. Items assess different aspects of maternal feelings and behaviors toward the infant such as the emotional connection, positive and negative feelings, and the overall relationship between mother-infant. Higher scores suggest poorer bonding or greater maternal distress, and lower scores indicate a stronger bond and healthier emotional connection. In analysis, total score (sum score of all items; range 0–125) will be used.

A short questionnaire addressing lifestyle behaviours (smoking, alcohol use), physical activity, dietary and screen time behaviours are applied. The physical activity and dietary questions are assessed using a modified version of a questionnaire developed and validated by the Swedish National Board of Health and Welfare [63]. Key dietary behaviours that are being assessed are: intakes of fruits and vegetables, sweetened beverages and snacks. Physical activity will be measured using four items on leisure time and occupational time prior to pregnancy, as well as time spent on moderate- and vigorous physical activity, sedentary behaviour and screen time during the last seven days during pregnancy. The collected data on dietary and physical activity behaviours will be summarised and analysed as in our previous research [64, 65].

In addition, information on obstetric and neonatal outcomes will be collected as continuous variables or incidence from medical records including its registries (pregnancy registry and Obstetrix, ORACLE Cerner, Sweden). Examples include birth experience, gestational weight gain, caesarean section, infant birth weight and length and incidence of large-for gestational-age infant (birth weight > 90th percentile for gestational age and gender).

### Data analysis

All statistical analyses will be conducted according to a pre-specified analysis plan. Descriptive statistics will be used to summarize baseline data including participant characteristics and baseline values. Continuous variables will be presented as means and standard deviations (or medians and interquartile ranges where more appropriate), and categorical variables as counts and percentages. Statistical significance will be evaluated at a two-sided α-level of 0.05, and 95% confidence intervals will be reported.

The primary analysis will examine between-group differences in psychological resilience at time-point 2 (8–9 weeks postpartum). A linear regression model will be fitted with psychological resilience at time-point 2 as the dependent variable and intervention group (HM4MH-app vs. active control app) as the independent variable. The model will be adjusted for parity (nulliparous vs. multiparous) and baseline value. The other stratification variable, site, will not be included in the model as this variable is not deemed to impact the intervention and outcome and was considered in the randomization to achieve balanced recruitment per site. After careful inspection of baseline characteristics, further variables as e.g. education may be included in the model to adjust for potential confounding caused by baseline imbalances between the study groups. The estimated mean difference between groups, with corresponding 95% confidence intervals and two-sided p-values, will be presented.

Secondary analyses will assess group differences at time-point 2 for depressive symptoms and psychological wellbeing using equivalent linear regression models. Exploratory outcomes (self-compassion, parental bonding during pregnancy and postpartum, fear of childbirth, and health behaviours) as well as obstetric and neonatal outcomes will be compared between groups using linear or logistic regression models, depending on the distribution and scale of outcomes.

To examine trajectories over the three time-points (baseline, T1, T2) and to test potential group-time interactions, linear mixed effects models will be used. Models will include fixed effects for time, group, and the interaction between group and time, as well as random intercepts for participants.

A pre-specified subgroup analysis will explore whether intervention effects differ by parity (nulliparous vs. multiparous). An interaction term between intervention group and parity will be included in the regression model to test for effect modification. If a statistically significant interaction is detected, results will be presented stratified by parity group. Furthermore, exploratory analyses will examine whether effectiveness varies by level of app engagement. Engagement will be quantified using objective usage metrics (composite score based on app usage time, number of logins, number of themes read and videos watched) and women will be categorized into quartiles (low to high engagement). Interaction terms between intervention group and engagement quartile will be included to assess effect modification. Statistically significant interactions will be followed by stratified analyses describing effect magnitude and direction. These analyses will be considered exploratory and hypothesis-generating, recognizing that app engagement may both mediate and modify intervention effects.

Any potential impact of missing data will be assessed in two steps. First, the missing data mechanism will be studied by investigating summary statistics of characteristics in completers and non-completers. This step will also include a drop-out analysis. Second, if missing at random can be assumed, sensitivity analyses may be conducted employing multiple imputation using chained equations with comparison to complete-case analyses.

If violation of the linear regression assumptions are detected, truncated linear regression or generalized linear regression will be considered and used if found more appropriate.

All statistical analysis will be conducted using appropriate statistical software such as SPSS (version 30.0 or higher), or R (version 4.5.2 or higher).

## Discussion

This randomized controlled trial will evaluate the effectiveness of a digital intervention (the HM4MH-app) aiming to enhance psychological resilience among women during the perinatal period. The intervention is designed to be health promotive, employing a salutogenic and universal approach, targeting a large proportion of women during the perinatal period.

The universal nature of the intervention increases its public health relevance and potential for population-level implementation but may reduce effect sizes compared to studies targeting high-risk individuals. This trade-off is intentional, consistent with the trial’s aim to evaluate a scalable, upstream health promotive strategy. Recruitment from a broad, diverse cohort of pregnant women may also enhance generalizability and ecological validity.

We have chosen resilience as the primary outcome since it has been identified as a changeable protective factor that may buffer against emotional distress during pregnancy and the postpartum period [23, 28]. By focusing on building psychological resources proactively, this trial contributes toward promoting mental health strategies within routine maternity care. Furthermore, it is also relevant to note that we have chosen a rather comprehensive approach for trial outcomes with both secondary and exploratory outcomes in order to reflect the multifaceted nature of psychological adaptation during the perinatal period. For example, fear of childbirth is a known predictor of negative birth experiences [66], while self-compassion has been associated with improved emotion regulation and lower postpartum distress [67]. Moreover, attachment measures allow for the assessment of the intervention’s impact on emerging parent-infant bonds, which are foundational for long-term child development and mental health for the mother. Thus, by including a broad array of outcomes, the study may offer a more thorough understanding of the pathways of a possible intervention effectiveness.

An active control app with focus on lifestyle behaviours was selected to control for nonspecific digital engagement effects, contributing to the trial’s internal validity [68, 69]. For example, factors such as time spent on using a pregnancy app per se or the general novelty of using a digital tool may be controlled for by matching both groups in format and delivery mode [69]. The two apps are delivered by the same digital platform with shared layout and other technical features. The design thus allows for the isolation of any potential “active ingredient” of the mental health content within the intervention app. Furthermore, the active control design may reflect a more ecologically valid comparison. Given the proliferation of mobile wellness apps, including for the perinatal period, an active-control design allows us to evaluate whether the mental health app delivers benefits beyond what might be achieved through general app engagement. Ultimatelly, this may yield more meaningful insights for potential real-world implementation within maternity health care in the future.

Although digital interventions holds great promise in their scalability and access, they present challenges related to engagement and attrition, particularly in longer interventions [70]. In addition, engagement is particularly important when assessing the effectiveness of stand-alone and self-guided digital interventions. Engagement was considered during intervention development in terms of app functions (e.g., reminder notifications, in-app reminders) and features (e.g., a combination of visual and auditory content). In addition, both objective and subjective engagement will be assessed throughout the trial. With this comprehensive approach we hope to be able to better monitor and understand how participants interact with the intervention and how engagement may influence outcomes.

## Conclusion

If shown to be effective, the intervention could offer a low-cost, accessible approach to promoting psychological resilience, emotional wellbeing, and early parent-infant attachment. This could support a shift toward proactive mental health promotion within perinatal care and contribute to long-term outcomes for both parents and their children.

## Supplementary Information

Supplementary Material 1. Additional file 1: SPIRIT 2025 checklist items

Supplementary Material 2. Additional file 2: Overview of primary estimand attributes

## Data Availability

The datasets used and/or analysed during the current study are available from the corresponding author on reasonable request.

## Abbrevations

HM4MH: HealthyMoms4MentalHealth
CD-RISC-10: Connor-Davidson Resilience Scale
RPWB: Ryff Scales of Psychological Well-Being
EPDS: Edinburgh Postpartum Depression Scale
FOBS: Fear of Birth Scale
SCS-SF: Self-Compassion Scale—Short Form
PAI: The Prenatal Attachment Inventory
PBQ: Postpartum Bonding Questionnaire
