# Supplementary material for: Promoting psychological resilience during the perinatal period with a digital tool (the HealthyMoms4MentalHealth-app): a study protocol for a randomised controlled trial in Sweden
Source: BMC Public Health. 2026 Oct 3;26:2950. doi: 10.1186/s12889-026-26804-8 (PMC13660349; doi:10.1186/s12889-026-26804-8)
Supplement: Supplementary file 1 [file 12889_2026_26804_MOESM1_ESM.pdf]

Additional file 2: Overview of primary estimand attributes.

Additional file 2: Table including primary estimand attributes

**Primary estimand**	
Intervention	Intervention = HealthyMoms4MentalHealth-app with focus on increasing psychological resilience.<br>Control = HealthyMoms-app with focus on healthy lifestyle.
Population	Pregnant women over 18 years with live birth and no technical hindrance in using the app.
Variable (Endpoint)	CD-RISC 10 score on 0-40 scale at time-point 2.
Intercurrent events	No outcome measures at time-point 2.
Strategy	While on treatment: we will use time-point 1 outcomes to analyze time-point 2.
Summary measure	Mean difference in resilience score between groups.
